# MineVisual: A Battery-Free Visual Perception Scheme in Coal Mine

**DOI:** 10.3390/s25175486

**Published:** 2025-09-03

**Authors:** Ming Li, Zhongxu Bao, Shuting Li, Xu Yang, Qiang Niu, Muyu Yang, Shaolong Chen

**Affiliations:** School of Computer Science and Technology, China University of Mining and Technology, Xuzhou 221116, China; lmgyw@cumt.edu.cn (M.L.); baozx@cumt.edu.cn (Z.B.); niuq@cumt.edu.cn (Q.N.); ts24170137p31@cumt.edu.cn (M.Y.); ts24170047a31@cumt.edu.cn (S.C.)

**Keywords:** battery-free visual sensing, energy harvesting, energy-aware adaptive pruning, deep learning inference, underground coal mine safety

## Abstract

The demand for robust safety monitoring in underground coal mines is increasing, yet traditional methods face limitations in long-term stability due to inadequate energy supply and high maintenance requirements. To address the critical challenges of high computational demand and energy constraints in this resource-limited environment, this paper proposes MineVisual, a battery-free visual sensing scheme specifically designed for underground coal mines. The core of MineVisual is an optimized lightweight deep neural network employing depthwise separable convolution modules to enhance computational efficiency and reduce energy consumption. Crucially, we introduce an energy-aware dynamic pruning network (EADP-Net) ensuring a sustained inference accuracy and energy efficiency across fluctuating power conditions. The system integrates supercapacitor buffering and voltage regulation for stable operation under wind intermittency. Experimental validation demonstrates that MineVisual achieves high accuracy (e.g., 91.5% Top-1 on mine-specific tasks under high power) while significantly enhancing the energy efficiency (reducing inference energy to 6.89 mJ under low power) and robustness under varying wind speeds. This work provides an effective technical pathway for intelligent safety monitoring in complex underground environments and conclusively proves the feasibility of battery-free deep learning inference in extreme settings like coal mines.

## 1. Introduction

In recent years, significant breakthroughs in battery-free sensing technology have fundamentally transformed industrial monitoring paradigms. By leveraging diverse energy harvesting mechanisms [1]—including triboelectric nanogenerators (TENGs) [2,3,4], near-field communication (NFC) [5], and RF backscattering [6]—this technology achieves truly self-powered operation [7]. This paradigm eliminates the recurring costs and environmental burdens of battery replacement, enabling sustainable long-term deployment in challenging environments including forests, oceans, and underground mines.

While significant progress has been made in battery-free sensing for terrestrial and subaquatic environments [8], research specifically addressing the unique challenges of underground coal mines remains nascent. Within this domain, battery-free technology predominantly leverages the mine ventilation system as the primary source for energy harvesting [9]. As a core component ensuring coal mine safety, the ventilation system offers a technically compatible source of wind energy due to the shaft’s stable airflow environment (average wind speed 4 m/s, uniformly distributed, max 10 m/s) [10]. While prior works have demonstrated the feasibility of powering basic sensor nodes via shaft wind energy [11,12], the harsh underground environment—characterized by dust, humidity, and fluctuating airflow—often compromises stability and reduces energy harvesting efficiency [13,14].

In recent years, battery-free technology in mine safety monitoring has primarily focused on basic energy harvesting and communication mechanisms, and it has yet to advance into complex applications such as machine learning (ML) model inference on devices. For safety monitoring, battery-free sensors in coal mines must not only collect data but also process it locally to reduce transmission costs and conserve scarce energy. Despite advances in lightweight techniques like model compression [15] and layer-wise quantization [16] that reduce computational energy, real-time ML inference on underground battery-free devices remains a pressing challenge due to stringent power constraints in dynamic environments.

This paper addresses the feasibility of battery-free ML in underground coal mines. We propose MineVisual, a novel battery-free visual perception scheme. It harvests energy from mine ventilation airflow via a custom low-starting-torque wind turbine, eliminating wiring and batteries for reduced maintenance and sustainability. Microcontroller nodes and cameras capture imagery, processed by a lightweight ML model on-device. This enables real-time detection of safety hazards—personnel location, helmet compliance, and tunnel deformation—while minimizing data transmission and conserving energy. However, realizing this scheme confronts two primary challenges:

- How to achieve stable and efficient energy harvesting from the mine ventilation system to power computation-intensive visual sensing under fluctuating wind conditions;
- How to design a lightweight ML model capable of dynamically adapting its computational complexity in real-time to maintain accuracy under constrained and variable power availability.

To tackle the first challenge, we designed a wind turbine with low-starting-torque DC motors, optimized for low-speed mine airflow, and combined with real-time power monitoring for dynamic regulation and stable operation. To address the second, we introduce **EADP-Net** (Energy-Aware Dynamic Pruning Network), a depthwise separable convolution-based model for energy efficiency. Unlike Meta-Pruning [17] or single-shot pruning [18], it features (1) an energy-state factor α (Equation (4)) adjusting pruning via a power surplus, thereby preventing accuracy drops; and (2) hierarchical-layer neuron optimization. This reduces FLOPs by 26.3–70.3% while maintaining 68.6–91.5% accuracy across energy states.

Accordingly, we rigorously evaluate two critical dimensions of MineVisual:

- The performance of lightweight deep neural network (DNN) models (including accuracy, inference latency, and energy efficiency) when executing critical safety monitoring tasks in the resource-constrained underground coal mine environment;
- The feasibility and effectiveness of the energy-aware adaptive pruning mechanism on ultra-low-power microcontroller platforms under real fluctuating power.

By designing an end-to-end workflow from energy harvesting to on-device inference, we tested accuracy and power consumption dynamically across dynamically varying energy conditions. The results show that optimized wind harvesting combined with the EADP-Net model enables complex visual DNN inference on battery-less devices. This establishes a pathway for sustainable, intelligent mine monitoring and proves adaptive low-power deep learning in extreme environments.

Compared to other battery-free sensing methods [9,19,20,21], as shown in Table 1, MineVisual is more capable of performing complex calculations and long-term deployment in coal mining environments. It is powered by wind turbines and combines an energy-aware dynamic pruning model (EADP-Net) to maintain high visual inference accuracy and energy efficiency under fluctuating energy conditions, enabling true battery-free edge intelligence for complex visual tasks such as personnel safety compliance monitoring.

The paper is organized as follows: Section 2 details scheme design; Section 3 presents energy harvesting and EADP-Net; Section 4 validates experiments; Section 5 concludes.

## 2. Scheme Overview

**Figure 1:**
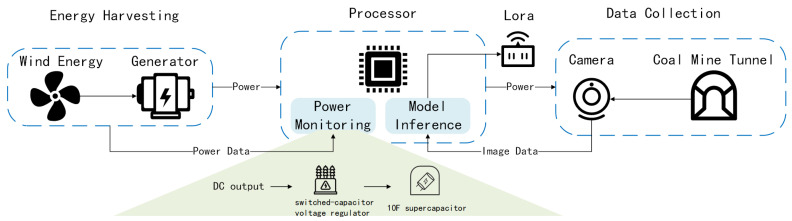
Working process of the battery-free visual perception scheme in coal mines.

In this section, we outline the design of the proposed battery-free visual perception scheme, MineVisual, specifically engineered for the demanding environment of underground coal mines. As depicted in Figure 1, MineVisual integrates two core components: (1) a custom wind energy harvesting module utilizing a low-starting-torque DC generator to efficiently capture ventilation airflow and convert it into electrical power, and (2) the EADP-Net, a lightweight deep neural network optimized for visual monitoring tasks under severe power constraints. The EADP-Net model incorporates a novel dynamic energy-aware pruning mechanism. This mechanism continuously monitors the real-time output power from the energy harvester and dynamically adjusts the network’s computational complexity in response to the current energy state. By pruning the network to reduce complexity and selectively disabling specific layers or neurons, the model adapts to fluctuating power availability while maintaining high inference accuracy.

The deployment of the MineVisual scheme within the operational underground coal mine environment follows a structured two-phase approach: offline training and underground inference.

**Offline Training Phase:** In this preparatory stage, video footage and images depicting personnel, equipment, and mine scenes are captured underground using the designated camera sensors. This raw visual data undergoes preprocessing steps, including image reshaping and normalization, to standardize the input format for model training. Subsequently, the EADP-Net model is subjected to optimization techniques, specifically network-layer pruning and neuron pruning, to reduce its structural complexity. This is followed by model quantization and compression, significantly diminishing the model’s storage footprint and computational demands. The outcome of this phase is a highly optimized, lightweight model specifically tailored for deployment on resource-constrained, low-power microcontrollers.

**Underground Inference Phase:** Upon deployment, the scheme harnesses energy from the custom wind turbine module and potentially other ambient sources to power the sensing and computation nodes. Concurrently, camera-equipped sensor nodes capture real-time visual data from the mine environment. Acquired images are immediately preprocessed (reshaping and normalization) to match the model’s input requirements. These preprocessed images are then fed into the lightweight EADP-Net model for on-device inference. Crucially, leveraging the integrated dynamic energy-aware pruning mechanism, the model autonomously adjusts its computational complexity (i.e., the extent of active layers and neurons) in real-time based on the instantaneous power output from the energy harvester. This dynamic adaptation ensures sustained inference accuracy and maximizes energy efficiency across the fluctuating power conditions endemic to mine ventilation systems. Finally, the model outputs its prediction results (e.g., classifications or detections), enabling real-time monitoring and the generation of critical alerts concerning personnel safety (location, PPE compliance) and infrastructural integrity (tunnel deformation).

## 3. Scheme Design

### 3.1. Energy Harvesting in Coal Mine

To achieve sustainable battery-free operation in underground coal mines, we propose a turbine-based rotational energy harvesting module that converts the kinetic energy of the mandatory ventilation airflow into electrical power. Although ventilation airflow is generated by powered fans, direct electrical coupling to industrial power lines is prohibited by mine safety standards (e.g., Safety requirements for mining main ventilation fan system [22]). Wind energy harvesting provides intrinsic isolation, eliminating risks of high-voltage exposure and simplifying deployment. A miniature wind turbine, installed within the mine’s ventilation ducts, directly drives a low-starting-torque DC motor, generating electrical energy without intermediate AC–DC conversion, thereby minimizing energy losses. The harvested energy is continuously monitored by an integrated power management unit, which dynamically regulates and allocates the available power to ensure a sufficient supply under varying operating conditions. The overall scheme architecture comprises three functional modules: energy harvesting, data collection, and processing. Electrical energy from the harvesting module is first delivered to the central processor. Once the processor detects that the capacitor voltage exceeds a predefined threshold, it activates the imaging subsystem to capture real-time underground imagery. The acquired images are then transmitted to the processor, where a lightweight, on-device model performs inference and analysis. Physical separation between the imaging module and the processor enables independent transmission paths for power and data via short-range cabling, improving both power stability and system responsiveness. Among those, the DC output is stabilized via a switched-capacitor voltage regulator (3.3 V output, 85% efficiency) and buffered by a 10F supercapacitor. This ensures sustained operation during wind lulls (<2.7 m/s), enabling cold-start at 3.6 V. The scheme activates only when the capacitor voltage exceeds 3.0 V, preventing undervoltage lockout. Furthermore, the scheme design leverages the periodic fluctuation characteristics of mine ventilation airflow to establish a flexible harvest–wake–sense–infer operational loop, thereby achieving intermittent yet autonomous functionality in the absence of a continuous power supply.

### 3.2. Energy-Aware Model Pruning Mechanism

**Figure 2:**
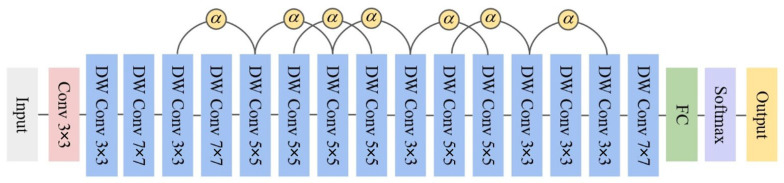
Energy-aware dynamic pruning network.

**Figure 3:**
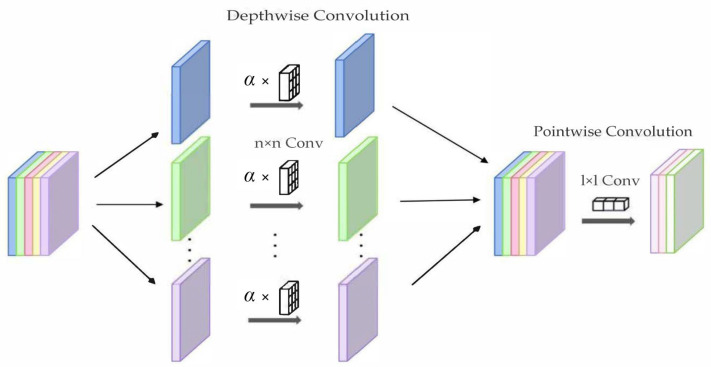
Energy-aware optimized depthwise separable convolution block.

Building upon the energy harvesting foundation, we now focus on designing an efficient, lightweight, and low-power deep learning model tailored for underground monitoring tasks. To address the stringent low-power inference requirements in coal mines, we propose the EADP-Net (Energy-Aware Dynamic Pruning Network). The overall architecture of EADP-Net is depicted in Figure 2. EADP-Net incorporates the design principles of established lightweight neural networks [23,24], utilizing depthwise separable convolution (DSC) as its fundamental building block [15]. By decomposing standard convolution into depthwise and pointwise convolutions, DSC significantly reduces computational complexity, model parameters, and memory footprint, thereby enhancing computational and energy efficiency. Crucially, we augment the DSC module with a novel energy-aware mechanism. This mechanism dynamically adjusts the processing complexity within the depthwise convolution stage based on the scheme’s real-time energy state. Specifically, it modulates the feature channel scaling factors (α in Figure 3) in response to the instantaneous power availability, enabling fine-grained control over computational resource allocation. This real-time adaptation optimizes energy consumption while maintaining task performance, ensuring efficient operation on resource-constrained underground hardware platforms. The structure of this enhanced energy-aware DSC module is illustrated in Figure 3. This design allows the network to adaptively adjust its computational load under varying energy conditions, enhancing scheme stability and efficiency while achieving the core objective of low-energy deep learning inference. Furthermore, to address the significant challenge of unstable power generation caused by wind speed fluctuations and to achieve further reductions in computational demand and energy consumption, we introduce a complementary energy-aware model pruning mechanism. This mechanism optimizes computational resource utilization by dynamically adjusting the number of active neural network layers and neurons based on the current energy state. The energy state is derived in real-time from the wind turbine module’s power output (P), monitored continuously during scheme operation. The instantaneous wind power P is calculated as

(1) $$P=\frac{1}{2}\rho Av^{3}\eta_{1}\eta_{2},$$

where ρ is the air density, A is the swept area of the wind turbine blades $A=\pi r^{2}$, which is related only to the area of the blades r, v is the wind speed, $\eta_{1}$ denotes the energy conversion efficiency of the fan blades, and $\eta_{2}$ denotes the conversion efficiency of the generator motor.

#### 3.2.1. Network-Layer Pruning

The goal of network-layer pruning is to decide whether to remove certain network layers based on the current energy state. Assuming that the total number of layers in the network is L, we can measure the importance of layer l by calculating its $L_{2}$ norm for the weight matrix $W_{l}$, which contains all the connectivity parameters of the layer. The $L_{2}$ paradigm number represents the overall size of the weights, and a larger $L_{2}$ norm usually means that the weights of that layer contribute more to the network performance.

(2) $$|W_{l}{|}_{2}=\sqrt{\sum_{i=1}^{m_{l}}\sum_{j=1}^{n_{l}}W_{l}{\left(i,j\right)}^{2}},$$

where $m_{l}$ and $n_{l}$ denote the input and output neuron counts of layer l, respectively, and $W_{\left(i,j\right)}$ is the connection weight between the i-th input neuron and the j-th output neuron of the layer l. The $L_{2}$ norm measures the Euclidean magnitude of the weight tensor, with higher values indicating stronger contribution to feature representation and thus greater importance to network performance. Based on the ratio of the calculated norm to the sum of the $L_{2}$ norms of all layers ($\sum_{l=1}^{L}\|W_{l}{\|}_{2})$, we calculate the pruning ratio $p_{l}$ for each layer:

(3) $$p_{l}=\frac{\|W_{l}{\|}_{2}}{\sum_{l=1}^{L}\|W_{l}{\|}_{2}},$$

where $p_{l}$ denotes the importance ratio of the weight of layer *l* to the total weight. To determine whether to retain a layer under different energy states, we introduce the energy state factor α, which is related to the current wind power generation intensity P and the average power generation capacity $\bar{P}$.

(4) $$\alpha =\frac{1}{1+e^{-\lambda (P-\bar{P})}},$$

where *λ* is a hyperparameter used to control the influence of power on pruning intensity. Then, we dynamically adjust whether to retain each layer based on the pruning ratio $p_{l}$ and the current energy state factor *α*. Set a threshold τ. When $p_{l}$ is less than this threshold, prune the layer; otherwise, retain the layer.

(5) $$L^{\prime }=\sum_{l=1}^{L}\max\left(0,\min\left(1,\frac{p_{l}}{\tau \cdot \alpha }\right)\right),$$

#### 3.2.2. Neuron Pruning

The goal of neuron pruning is to selectively remove unimportant neurons based on their importance, thereby reducing computational load and energy consumption. Suppose that layer l has $N_{l}$ neurons. For the i-th neuron in layer l, its activation value $A_{l,i}^{\left(t\right)}$ in the t-th forward propagation can be obtained using the activation function f:

(6) $$A_{l,i}^{\left(t\right)}=f\left(Z_{l,i}(t)\right),$$

where $Z_{l,i}^{\left(t\right)}$ is the input signal of neuron i during the t-th forward propagation, which is usually a weighted sum:

(7) $$Z_{l,i}^{\left(t\right)}=\sum_{j=1}^{N_{l-1}}W_{l,i,j}A_{l-1,j}^{\left(t\right)}+b_{l}^{i},$$

where $W_{l,i,j}$ is the weight connecting the j-th neuron in layer l−1 to the i-th neuron in layer l, b is the bias term of the i-th neuron in layer l, and $A_{l-1,i}^{\left(t\right)}$ is the activation value of the j-th neuron in layer l−1 during the t-th forward propagation. We use the mean squared activation value (MSA) of the activation value of the i-th neuron in the layer l as a measure of its importance. This metric represents the average activation energy of the neuron across the training set. Neurons with consistently low activations (MSA close to 0) contribute minimally to downstream feature representations, indicating their potential redundancy and suitability for pruning.

(8) $$I_{l}^{i}=\frac{1}{T}\sum_{t=1}^{T}{\left|A_{l,i}^{\left(t\right)}\right|}^{2},$$

where $A_{l,i}^{\left(t\right)}$ is the activation value of the i-th neuron in the layer l during the t-th forward propagation, and T is the number of time steps in the forward propagation, which is typically equal to the number of training samples or the number of batches of data. Based on MSA values of neurons (defined in Equation (8)) and the energy state factor α introduced above, we establish a dynamic threshold T to prune neurons exhibiting lower importance.

(9) $$T=\alpha \cdot \frac{1}{N_{l}}\sum_{i=1}^{N_{l}}I_{i}^{l},$$

#### 3.2.3. Pruning Strategy

We design a hierarchical pruning strategy that dynamically adapts neural network complexity to real-time energy availability, governed by the energy state factor α:

**High-Energy State (*p* ≥ 90 mW):** Under sufficient wind power, the scheme deploys the full network architecture without pruning. This maximizes inference accuracy for critical tasks (e.g., personnel detection, helmet compliance) by retaining all layers and neurons.

**Medium energy state (40 mW ≤ *p* < 90 mW):** When the wind speed is moderate and the harvested energy is usable but not abundant enough to fully support all computations, the scheme automatically prunes some neural network layers and neurons dynamically. Specifically, we prioritize pruning neurons and layers that contribute less to task performance. This reduces the computational burden and power consumption without significantly compromising model accuracy. This dynamic pruning process is based on the importance of the weights within the layers and neurons. Furthermore, the pruning ratio is dynamically adjusted by an energy-aware mechanism that monitors computational demand in real-time, aiming to achieve a reasonable balance between performance and energy efficiency.

**Low-energy state (*p* < 40 mW):** Under severe energy constraints, aggressive pruning retains only core computational pathways. To ensure that basic task execution can still be maintained under ultra-low-power conditions, we increase the pruning intensity, reducing the number of layers and neurons in the model. At this stage, the pruning strategy prioritizes retaining core computational capacity, ensuring the model can still accomplish the most critical tasks when energy resources are severely limited.

This energy-state-driven pruning strategy optimally allocates limited power resources to computational demands. By reducing redundant operations during low-energy intervals and maximizing accuracy during high-energy windows, the scheme achieves sustained task performance (85.2% Top-1 accuracy at 6.89 mJ) while enhancing operational sustainability in volatile mine environments.

The complete energy-aware pruning workflow is formalized in Algorithm 1, which dynamically adjusts model complexity based on real-time power *P*, average power $\bar{P}$, and energy state factor α.
**Algorithm 1.** Low-Energy Consumption Model Based on Energy Awareness1:**Input:** *P* (real-time wind power), $\bar{P}$ (average wind power over a time window), *λ* (scaling factor for energy state sensitivity), *N* (total network layers), *M* (total neurons per layer), trainSet, valSet2:**Output:** Optimized neural network model3:*M ←* initial-DNN(*N*, *M*)//*Construct lightweight DNN with N layers and M neurons per layer*4:$\alpha \leftarrow \frac{1}{1+e^{-\lambda \left(P-\bar{P}\right)}}$//*Define energy state factor*5:*β ←* 1//*Set initial pruning factor*6:**for** each training iteration **do**7: *β ← α/*/*Set pruning factor based on energy state*8: Prune *β·N* layers with least importance//*Perform layer pruning based on importance ranking*9: Prune *β·M* neurons per layer with least contribution//*Perform neuron pruning*10: Fine-tune the pruned model *M* on trainSet//*Fine-tune the model*11:**end for**12:Evaluate the pruned model *M* on valSet//*Evaluate the model on the validation set*13:**if** performance degradation exceeds threshold **then**14: Roll back the last pruning operation//*Restore the previous state*15:**end if**16:Adjust energy state factor dynamically if *P* changes significantly//*Recalculate pruning factor if P fluctuates*17:**repeat**18: Repeat steps 3–9//*Continue pruning and adjusting*19:**until** convergence or significant energy state change **return** optimized model *M/*/*Output the final optimized model*

### 3.3. Battery-Free Inference in Mine

**Figure 4:**
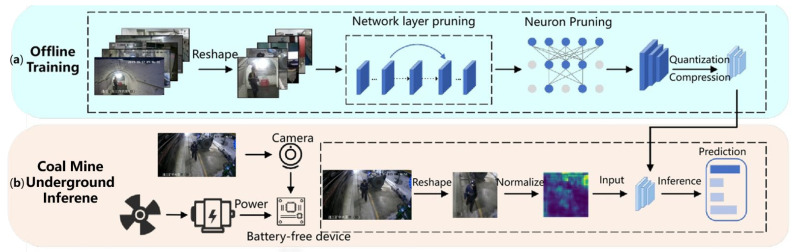
Offline training and on-board inference. (**a**): offline training: dataset preprocessing → model pruning → quantization/compression; (**b**): on-board inference: wind energy harvesting → image capture → EADP-Net adaptive inference → safety alerts.

To address the critical need for real-time hazard detection in high-risk underground mines, we implement on-device inference to eliminate cloud dependency and transmission delays. This approach leverages local computational resources to achieve ultra-low-latency analysis (<330 ms total runtime) essential for safety-critical responses. Furthermore, the network environment in underground coal mines is often unstable or even non-existent in certain areas. Relying on cloud-based inference cannot guarantee reliable scheme operation, whereas on-board inference functions independently without requiring network connectivity. Additionally, on-board inference reduces the energy consumption associated with data upload and leverages local computational resources to achieve efficient, low-power inference. To achieve this objective, we first conduct offline training using efficient hardware resources. Subsequently, to accommodate the computational and storage constraints of low-power hardware platforms, the model undergoes compression and quantization. Finally, the optimized model is deployed on a microcontroller, enabling battery-free, low-power inference underground. The overview of offline training and on-board inference is shown in Figure 4.

**Offline Training:** To ensure robust deployment on low-power underground devices, we adopted a multi-stage training approach. Initial training leveraged Mini-ImageNet [25] and Tiny-ImageNet [26] to establish baseline accuracy under controlled conditions. Subsequently, to maintain accuracy in the complex underground environments characterized by insufficient lighting and dust interference, the CUMT-CMUID coal mine image dataset [27] and CUMT-HelmeT safety helmet dataset [28] were used. These datasets were specifically collected and constructed for coal mine operational environments to enhance the robustness of critical visual tasks: personnel detection, safety compliance verification, and tunnel deformation monitoring. To mitigate challenges from low-light conditions and airborne particulates, we implemented targeted preprocessing. First, we augmented the dataset diversity through techniques such as rotation, flipping, cropping, and scaling to simulate image variations under different viewpoints and illumination conditions. We also adjusted image brightness and contrast to accommodate potential underground lighting fluctuations. Furthermore, we introduced noise to improve the model’s resilience to environmental interference, thereby enhancing its generalization capability. Second, to accelerate training and improve model stability, we normalized the images by scaling pixel values to the range [0, 1] and standardizing each channel to have zero mean and unit variance. This effectively mitigates the negative impact of inter-image lighting variations during training, promoting faster convergence. Finally, considering the constrained computational capabilities of low-power underground devices, we resized the images to a smaller fixed resolution (128 × 128 pixels). This reduction in dimensionality significantly decreases the computational load and memory footprint, thereby improving the inference speed to meet the resource constraints of the target hardware. Model training employed cross-validated workflows with tuned loss functions and optimizers, ensuring generalization across mine topographies while preventing overfitting. This pipeline balances accuracy with the stringent resource constraints of target hardware.

**Quantization:** Post-training quantization is applied to the optimized deep neural network (DNN) model to enhance deployability on ultra-low-power microcontrollers. This process converts the model’s floating-point weights and activation values into low-precision integers, significantly reducing memory footprint and computational latency with minimal precision loss. After converting the trained model to TensorFlow Lite format, we employ post-training quantization via STM32’s X-CUBE-AI toolchain to perform the quantization [29]. The tool automatically analyzes the distributions of weights and activations, adjusts quantization parameters, and calculates scaling factors to minimize precision loss. Finally, it generates an optimized quantized model suitable for the microcontroller, prepared for subsequent deployment and inference.

**Deployment:** Following model optimization, the quantized deep learning model is deployed onto the microcontroller to enable real-time inference for underground safety tasks. To support dynamic model pruning under varying energy states, we integrated a power monitoring subsystem that directly interfaces with the wind turbine’s output. This subsystem employs a precision power sensor (INA226AIDGSR manufactured by Texas Instruments from Dallas, TX, USA) coupled with an analog-to-digital converter (ADC) to continuously sample current and voltage data. The acquired measurements are converted into real-time power values (*p*) within the microcontroller, creating a closed-loop feedback mechanism. These instantaneous power readings directly trigger the EADP-Net’s adaptive pruning module, which dynamically configures the network’s active layers and neurons based on the current energy state. Inference results are transmitted via LoRa (Semtech SX1276 manufactured by Semtech from Camarillo, CA, USA) at 10 dBm output power (as shown in Figure 1). Each transmission consumes 14.2 mJ (20-byte payload), scheduled only when there is a supercapacitor charge > 80% to avoid scheme shutdown. This design demonstrates less than 1% data loss during 500 m tunnel testing [30]. This integrated approach ensures stable, low-power inference operations in underground coal mines—even during significant wind energy fluctuations—while maintaining critical safety monitoring capabilities.

## 4. Experiments

### 4.1. Wind Power Generation Device

**Figure 5:**
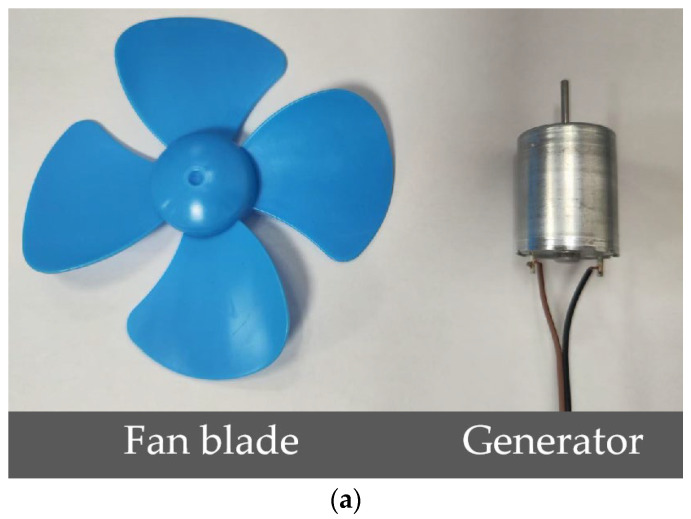

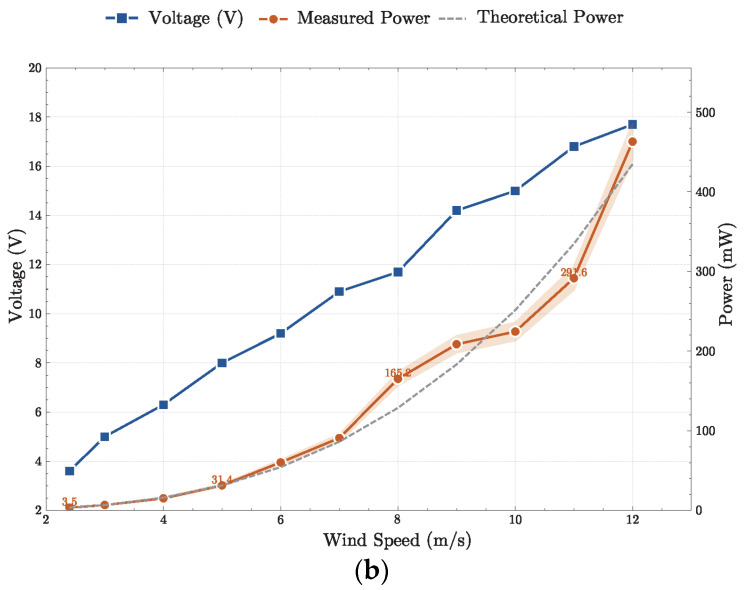
Wind power generation device and its power curve with wind speed. (**a**): The fan blade and generator of the wind power generation; (**b**): voltage–power curve with wind speed and supercapacitor charging profile.

The wind energy harvesting module employs a custom turbine with 50 mm radius blades coupled to a DC generator (12 mm radius, 34 mm height, 13 mm output-shaft height; see Figure 5a). Wind conditions were generated by a 122 W industrial ventilation fan (rated speed 2000 r/min, airflow 2650 m^3^/h, maximum wind speed 20 m/s). For the turbine, the measured no-load voltage shows a cut-in wind speed of 2.7 m/s (3.6 V), and rises approximately linearly from 5.0 V to 11.7 V over 3–8 m/s. To further characterize the wind energy harvesting module’s power output, we measured its delivered power at different wind speeds. The results (see Figure 5b) indicate that in the 2.7–4.0 m/s range, the output power increases slowly with wind speed, reaching at most 15.2 mW; in the 5–6 m/s range, the output power rises rapidly from 31.4 mW to 60.3 mW, approaching the power consumption level of the sensing node; and in the 7–8 m/s range, the growth accelerates further, with a maximum measured output of 165.2 mW. In the wind speed range of 1.5–3.0 m/s, the scheme can meet the minimum operating voltage required by the camera and the microcontroller. The system integrates the wind power generation module with the power management unit (BQ25570 manufactured by Your Cee from Shenzhen, China) in a single package, incorporating a 10F supercapacitor (BCAP0010 P300 X11 manufactured by Maxwell Technologies from San Diego, CA, USA) for temporary energy storage, ensuring a stable power supply even under fluctuating wind speeds. Based on this power curve, the voltage threshold control module startup logic can be set to ensure that the system only enters operational mode under safe energy conditions, thereby preventing abnormal interruptions or false detections caused by low voltage.

### 4.2. Model Performance Evaluation

#### 4.2.1. Benchmark Performance and Energy Grading

To establish a performance benchmark, we first evaluated the offline-trained model’s accuracy under stable high-power conditions (simulating optimal wind speeds). This ensures that the trained model can run properly on the STM32 development board under these conditions and achieve high accuracy as a benchmark performance. Next, prior to the model pruning experiments, it was necessary to define the wind speed ranges corresponding to different energy states because the power output of the wind power generation scheme is directly impacted by wind speed, an unavoidable factor in practical applications. Fluctuations in wind speed led to variations in power output, requiring real-time monitoring of wind speed and power generation combined with the actual power supply requirements of the STM32 development board to appropriately adjust the neural network complexity and model operating state. We established distinct energy states to reflect different operational conditions of the wind power scheme: high wind speeds enable the scheme to provide ample power, suitable for running compute-intensive tasks; conversely, low wind speeds result in reduced power output, necessitating decreased model complexity to lower computational consumption, thereby extending scheme runtime while maintaining basic task execution capability. We defined three discrete energy states based on real-time generator output (*p*), as illustrated in Table 2.

#### 4.2.2. Dynamic Pruning Strategy Validation

To rigorously validate the efficacy of the energy-aware pruning mechanism, we evaluated network-layer pruning, neuron pruning, and joint-layer neuron pruning across three distinct energy states (high: *p* ≥ 90 mW; medium: 40 mW ≤ *p* <90 mW; low: *p* < 40 mW) using the Mini-ImageNet dataset [25]. The experiments aimed to optimize computational performance by dynamically adjusting model complexity under varying energy conditions while maintaining inference accuracy and efficiency. As shown in Table 3, under high-energy conditions, we first benchmarked the performance of the original model, recording its parameter count, floating-point operations (FLOPs), memory footprint (for SRAM and Flash), and inference accuracy (Top-1 and Top-5), and thereby establishing baselines for subsequent pruning. The baseline model achieved 79.7% Top-1 accuracy with 39.59 K parameters and 1610.56 KB Flash usage. For medium-energy states, we applied a moderate pruning strategy, removing selected network layers and neurons to reduce computational load while minimizing the impact on inference accuracy. And computational load was reduced by 26.3% (to 1402.1 MFLOPs) while retaining 78.6% Top-1 accuracy. Under low-energy constraints, we implemented more aggressive pruning, cutting additional network layers and neurons to adapt to the constrained energy supply. Aggressive joint pruning further minimized resource consumption (979.56 KB Flash, 68.6% Top-1 accuracy) while reducing inference energy by 70.3% compared to high-power operation. Critically, experimental results demonstrate a tunable accuracy–efficiency tradeoff where energy savings scaled proportionally to pruning intensity without catastrophic accuracy collapse. These results confirm that our energy-adaptive approach dynamically optimizes computational resource allocation in response to power fluctuations—a critical capability for sustainable operation in volatile mine environments.

**Figure 6:**
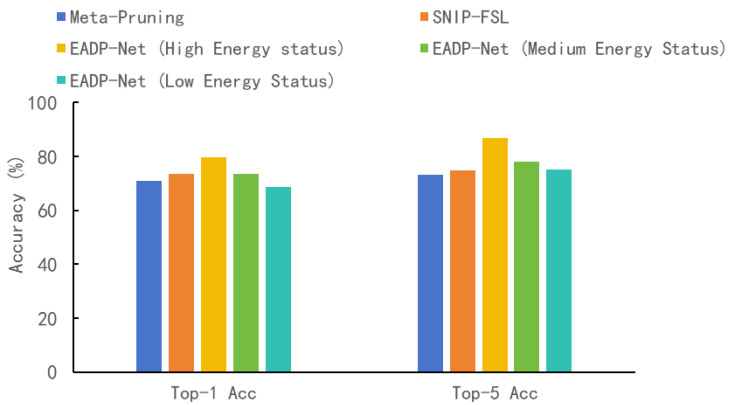
Comparison experiments of different pruning strategies on the Mini-ImageNet dataset.

Figure 6 shows the performance of several pruning strategies on the Mini-ImageNet dataset [25], measured by Top-1 and Top-5 accuracy. Under high-energy conditions, EADP-Net achieves a Top-1 accuracy of 79.7% and a Top-5 accuracy of 86.7%, outperforming Meta-Pruning [17] (70.8%/73.3%) and SNIP-FSL [18] (73.5%/74.8%). Specifically, EADP-Net also demonstrates good performance when the energy budget is reduced. In the medium-energy states, its Top-1 accuracy reaches 73.6% and Top-5 accuracy reaches 78.2%, matching or surpassing the comparison methods in terms of Top-5 accuracy and maintaining effective competitiveness in terms of Top-1 accuracy. Even under the low-energy conditions, EADP-Net maintains a reasonable Top-1 accuracy of 68.6% and Top-5 accuracy of 75.3%, demonstrating its robust performance under strict resource constraints. These results indicate that EADP-Net offers a favorable tradeoff between accuracy and efficiency across different operating points, making it suitable for deployment scenarios with varying energy budgets.

#### 4.2.3. Robustness Evaluation Across Heterogeneous Datasets

To assess the model’s generalization capabilities under domain shift, we evaluated the model’s performance across different datasets: Mini-ImageNet [25] (with 60,000 images divided into 100 classes), Tiny-ImageNet [26] (with 100,000 images divided into 200 classes), and our purpose-built coal mine dataset. The coal mine dataset is constructed based on the CUMT-CMUID [27] underground coal mine image dataset and the CUMT-HelmeT [28] underground safety helmet detection dataset, utilizing high-resolution images captured by KBA12(B) intrinsically safe alarm cameras in multiple underground coal mines. It encompasses diverse scenarios such as underground tunnels, workshops, and coal mine conveyor belts. The high-resolution images typically contain substantial background information irrelevant to the task, imposing significant computational burdens on low-power devices. Consequently, to accommodate the resource constraints of the STM32 development board, we cropped the images to retain task-relevant regions, such as personnel, equipment, and tunnels. The resulting cropped coal mine dataset comprises 1000 images, each with a resolution of 128 × 128 pixels, categorized into four classes: Person, Helmet-person, Tunnel, and Belt-foreign matter. This approach reduces computational load while enhancing image processing efficiency, enabling the dataset to better suit the processing requirements of low-power devices.

**Figure 7:**
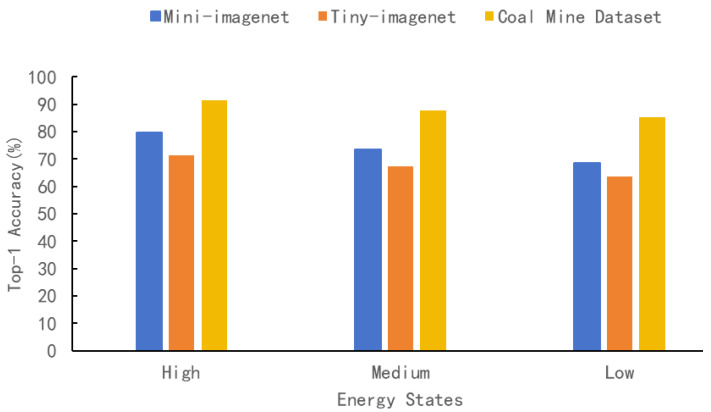
Inference accuracy of MineVisual on three different datasets.

In order to prove the inference accuracy of MineVisual under different energy modes, the coal mine dataset was evaluated together with Mini-ImageNet and Tiny-ImageNet. As quantified in Table 4 and Figure 7, under high-energy conditions, the inference accuracy of MineVisual achieved a Top-1 accuracy of 91.5% on the coal mine dataset, outperforming Mini-ImageNet (79.7%) and Tiny-ImageNet (71.2%), and thus indicating the strong adaptability and learning capability of our scheme for this specific condition. As the energy state decreased, the model’s accuracy gradually declined. However, even under low-energy conditions, the inference accuracy of MineVisual maintained a Top-1 accuracy of 85.2% on the coal mine dataset, demonstrating a robust performance for underground coal mine tasks.

#### 4.2.4. Quantization Impact on Deployment Efficiency

To facilitate effective deployment of the trained model on an STM32 development board, we employed the X-CUBE-AI tool to apply quantization at FP32 (32-bit floating-point), FP16 (16-bit floating-point), and Int8 (8-bit integer) precision levels. We then evaluated the model’s accuracy, memory usage, and inference latency on the Mini-ImageNet dataset under different power states.

As shown in Table 5, the experimental results demonstrate that reducing the quantization precision significantly decreases both the model’s memory footprint and inference latency. For instance, under the high-power state, the FP32 quantized model exhibits the highest memory consumption (SRAM: 483.12 KB, Flash: 1610.56 KB), an inference latency of 357 ms in Mini-ImageNet (400 ms in the coal mine dataset), and a Top-1 accuracy of 79.7% in Mini-ImageNet (91.5% in coal mine dataset). Progressively lowering the quantization precision to FP16 and Int8 gradually reduces the memory usage and inference latency, while the accuracy experiences a slight decrease, yet remains reasonably good. Under the low-power state, the Int8 quantized model requires 250.17 KB of SRAM and 816.15 KB of Flash, achieves an inference latency of 193 ms in Mini-ImageNet (216 ms in coal mine dataset), and maintains a Top-1 accuracy of 66.2% in Mini-ImageNet (79.9% in coal mine dataset). These experiments validate that quantization effectively reduces memory requirements and inference latency while preserving acceptable performance levels under low-power conditions, thus demonstrating its efficacy for deploying deep learning models in resource-constrained environments.

#### 4.2.5. Ablation Study on Energy-Aware Pruning

To isolate the independent contribution of energy-aware pruning (EAP) from other optimizations such as quantization and depthwise separable convolutions (DSCs), we conducted controlled ablation experiments. Four configurations were considered: (A) DSC + FP32 without pruning, (B) DSC + FP32 with EAP, (C) DSC + INT8 without pruning, (D) DSC + INT8 with EAP. These experiments reveal the unique effect of pruning on accuracy, memory footprint, inference latency, and energy consumption.

The results in Table 6 demonstrate that quantization alone (A → C) reduces latency by 36.7% and Flash by 40.2% with a 5.1-point Top-1 accuracy drop. Pruning alone (A → B) reduces Flash by 34.4% (mid energy) with a 6.1-point Top-1 drop; aggressive pruning at low energy achieves a 39.2% Flash reduction with an 11.1-point drop. System-level measurements further show that, when combined with quantization, EAP reduces inference energy from 23.23 mJ (high) to 13.76 mJ (medium) and 6.89 mJ (low), while keeping end-to-end latency within 329–359 ms. These results confirm that EAP provides independent and tunable efficiency gains beyond quantization and DSC.

### 4.3. System-Level Energy Efficiency Assessment

**Figure 8:**
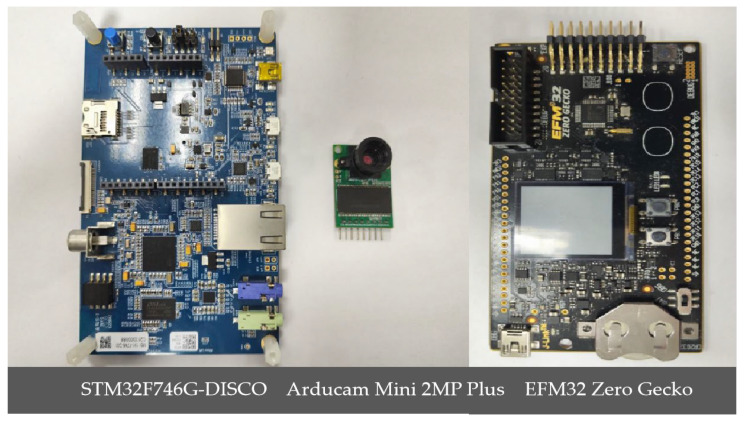
Hardware equipment for low-power reasoning in coal mines.

Figure 8 depicts the hardware equipment for low-power reasoning in coal mines. To evaluate the end-to-end energy efficiency of MineVisual under operational conditions, we measured the scheme’s power consumption during model inference using an STM32F746G-DISCO (manufactured by STMicroelectronics, made in Shenzhen, China) development board (320 KB SRAM/1 MB Flash) integrated with an Arducam Mini 2 MP Plus camera module (manufactured by Arducam, from Nanjing, China); an EFM32 Zero Gecko module (manufactured by Silicon Labs, from Austin, TX, USA) was employed for high-resolution power profiling. In order to balance energy and computational requirements, the low-power mine inference platform used an edge processor with neural network acceleration as the main controller, complemented by an infrared camera optimized for low-illumination imaging and a low-power LoRa radio for intermittent long-range telemetry, so that the overall scheme’s power budget is constrained below 2 W. For field deployment underground, all components are packaged within an explosion-proof, dustproof enclosure and installed using magnetic and snap-fit mechanisms for quick installation and removal on mine supports. Optical and mechanical front-end protections (a wind-driven dust shield and optical filter) are employed to mitigate coal dust contamination and improve image quality under weak lighting. The power subsystem is designed for plug-and-play autonomous operation. When the wind-driven generator produces sufficient voltage, the integrated power management unit permits self-excitation and the platform executes the complete cycle of data acquisition, on-device inference, and subsequent data transmission. This integrated hardware and measurement approach provides a realistic assessment of MineVisual’s energy performance and demonstrates feasibility for passive, low-power visual sensing in constrained underground environments. The model was quantized to INT8 to accommodate hardware constraints. Power metrics were evaluated across three energy modes, with key parameters defined as follows:

(1) **Inference Power**: Active power during model execution;
(2) **Inference Time**: Duration per inference pass;
(3) **Inference Energy**: Total energy consumed during inference (E_inf_ = P_inf_ × t_inf_);
(4) **Total Runtime**: End-to-end latency (image capture, preprocessing, inference);
(5) **Total Energy**: System-wide consumption (E_total_ = P_avg_ × t_total_).

As illustrated in Table 7, the high-power mode (*p* ≥ 90 mW) yields 102.8 mW inference power, 226 ms inference time, and 28.95 mJ total energy consumption at 329 ms runtime. Under medium-power (40 mW ≤ *p* < 90 mW), these reduce to 68.1 mW and 21.24 mJ (202 ms). Critically, the low-power mode (*p* < 40 mW) achieves 6.89 mJ inference energy (70.4% reduction vs. high power) and 13.49 mJ total energy despite an 8.1% runtime increase (359 ms) from non-inference overheads. Although the inference time decreases with lower-power modes, the time required for other stages (such as data sampling and preprocessing) increases, resulting in a slight overall increase in total runtime. This demonstrates MineVisual’s ability to maintain functionality during energy scarcity while reducing inference power by 65.3% versus the high-power mode. Despite a modest increase in inference time, overall energy efficiency improves, validating the effectiveness of quantization and power-aware mechanisms, and confirming that the energy generated by the wind power node meets the application requirements of our low-power device.

To provide a holistic view of the proposed system, we further summarize its system-level performance beyond the inference results. Table 8 highlights the end-to-end energy budget, energy harvesting range, communication protocol, functionality, payload characteristics, and detailed energy breakdown. This overview enables readers to better understand the practical feasibility and deployment conditions of the proposed self-powered LoRa sensing-inference system.

## 5. Conclusions

This paper introduces MineVisual, a novel battery-free visual perception system designed for underground coal mines, which enables sustainable deep learning inference under extreme energy constraints. By combining a customized wind energy harvester optimized for low-speed ventilation airflow and EADP-Net—a lightweight deep neural network employing depthwise separable convolutions—the system achieves substantial computational efficiency. An energy-aware adaptive pruning mechanism dynamically adjusts model complexity through layer-wise and neuron-wise pruning in response to real-time power variations, preserving high inference accuracy (e.g., 91.5% Top-1 under sufficient power) while significantly reducing energy consumption (as low as 6.89 mJ per inference under low power). The experimental results demonstrate robust performance across variable wind speeds (2.7–10 m/s) in critical safety applications such as personnel detection, helmet compliance monitoring, and tunnel deformation analysis. Although a slight decline in accuracy occurs under ultra-low-power conditions (e.g., 85.2% Top-1 at 35.7 mW), the system exhibits remarkable energy efficiency and operational resilience in harsh mining environments. This work establishes a foundational framework for self-adaptive, battery-free deep learning in resource-limited industrial settings, opening up new avenues for intelligent safety monitoring under extreme conditions. Practical challenges such as dust accumulation, mechanical wear, and intermittent wind availability remain to be addressed in future efforts, which will focus on enhanced energy harvester reliability, improved power management, supercapacitor integration, dust-proof designs, and further algorithm-level optimizations for better energy–accuracy tradeoffs.

## Figures and Tables

**Table 1:** Comparison with other battery-free sensing methods.

	Underwater Backscatter Imaging [19]	Underwater Battery-Free ML [20]	TESS [9]	TENG-SA [21]	MineVisual (Ours)
Primary Feature	Wireless underwater imaging	Marine/underwater environmental monitoring	Air quality monitoring in gold mines	Human motion monitoring and human–robot interaction (HRI)	Safety monitoring in coal mines
Energy Source	Underwater sound	Underwater sound	Wind energy	Mechanical energy	Wind energy
Communication Method	Acoustic backscatter	Acoustic backscatter	LoRa	Bluetooth	LoRa
Processing and Intelligence	On-board processing unit (FPGA) controls image capture, data packetization, and communication	Small CNNs trained offline, Keras2C implementation	A data acquisition module aggregates and compiles data from multiple sensors	LSTM-based network with adjustable complexity	On-device deep learning inference with a lightweight model (EADP-Net)
Key Performance	Average power: ~276 µW (active imaging) data rate: ~1 kbps	Total energy/inference: 5.40 mJ accuracy: ~63% (small sample dataset)	Starting windspeed: 0.32 m/s power density: 16.36 mW/m^2^ lifetime: >3 months	Output voltage range: 1.8–10.5 V; recognition accuracy: ~90%	Inference energy: 6.89 mJ (low power) accuracy: 68.6–91.5% (top-1)
Target Environment	Underwater environments	Underwater environments	Underground gold mines	Wearable, on-body applications	Underground coal mines

**Table 2:** Different energy states based on real-time generator output.

Energy State	Real-Time Power Output
High	*p* > 90 mW
Medium	40 mW < *p* < 90 mW
Low	*p* < 40 mW

**Table 3:** Comparative experiments conducted on network-layer pruning, neuron pruning, and joint network-layer and neuron pruning in three different energy states.

	Energy Status	Params	FLOPs	SRAM	Flash	Top-1	Top-5
		(M)	(M)	(KB)	(KB)	Acc (%)	Acc (%)
Original	High	0.41	39.59	483.12	1610.56	79.7	86.7
Pruning (Layer)	Medium	0.38	29.86	457.23	1402.1	78.6	83.2
	Low	0.36	21.17	387.2	1186.35	74.5	80.1
Pruning (Neurons)	Medium	0.39	31.47	468.53	1215.36	78.1	83.1
	Low	0.35	19.82	363.91	1076.54	73.2	79.7
Pruning (Layer-Neurons)	Medium	0.35	19.27	359.56	1056.35	73.6	78.2
	Low	0.28	15.26	312.78	979.56	68.6	75.3

**Table 4:** Inference accuracy of MineVisual on three different datasets.

Energy Status	Mini-ImageNet		Tiny-ImageNet		Coal Mine Dataset
	Top-1 (%)	Top-5 (%)	Top-1 (%)	Top-5 (%)	Top-1 (%)
High	79.7	86.7	71.2	76.5	91.5
Medium	73.6	78.2	67.2	71.4	87.7
Low	68.6	75.3	63.5	68.6	85.2

**Table 5:** Comparison experiments with different quantization accuracies.

Quant	Energy Status	SRAM (KB)	Flash (KB)	Inference Latency (ms)		Top-1 (%)	
				MINI-ImageNet	Coal Mine	MINI-ImageNet	Coal Mine
FP32	High	483.12	1610.56	357	400	79.7	91.5
	Medium	359.56	1056.35	289	324	73.6	87.7
	Low	312.78	979.56	248	278	68.6	85.2
FP16	High	356.17	1214.32	276	309	77.3	88.9
	Medium	327.71	968.13	266	298	76.5	85.2
	Low	294.11	912.74	215	241	68.1	82.7
Int8	High	287.5	963.12	226	253	74.6	86
	Medium	274.36	888.32	202	226	73.2	82.3
	Low	250.17	816.15	193	216	66.2	79.9

**Table 6:** Ablation experiment on energy-aware pruning.

Config	Quant.	Pruning	Top-1 (%)	Top-5 (%)	Flash (KB)	SRAM (KB)	Latency (ms)	Notes
A	FP32	✗	79.7	86.7	1610.56	483.12	357	Baseline-FP32 (High)
B-M	FP32	EAP (mid)	73.6	78.2	1056.35	359.56	289	Pure pruning (Medium)
B-L	FP32	EAP (low)	68.6	75.3	979.56	312.78	280	Pure pruning (Low)
C	INT8	✗	74.6	79.3	963.12	287.50	226	Quantization only (High)
D-M	INT8	EAP (mid)	69.5	72.3	631.81	213.97	183	(INT8 + Pruning, Medium)
D-L	INT8	EAP (low)	65.0	69.9	585.58	186.13	177	(INT8 + Pruning, Low)

**Table 7:** Energy consumption analysis for three different energy states.

Energy Status	Inference Power	Inference Time	Inference Energy	Total Runtime	Total Energy
	(mW)	(ms)	(mJ)	(ms)	(mJ)
High	102.8	226	23.23	329	28.95
Medium	68.1	202	13.76	332	21.24
Low	35.7	193	6.89	359	13.49

**Table 8:** System-level summary of the proposed self-powered LoRa sensing-inference system.

Metric	Value/Description
End-to-end energy per cycle	~30–35 mJ (including sensing, inference, and LoRa transmission)
Harvesting range	Self-sustainable at wind speeds ≥ 3.7 m/s (tested with micro-turbine energy harvester)
Wireless protocol	LoRa (868/915 MHz band), max payload 51 bytes per packet
Functionality	Image sensing (low-resolution grayscale), on-device EAP-Net inference, LoRa transmission of results
Payload size	~32 KB image before compression; inference results compressed to <1 KB for LoRa
Energy breakdown	Sensing: ~3–5 mJ; inference: ~6.9–23.2 mJ (depending on pruning level); transmission: ~5–7 mJ

## Data Availability

Data are available in a publicly accessible repository. Mini-ImageNet datasets: https://github.com/yaoyao-liu/mini-imagenet-tools, accessed on 2 May 2025; Tiny-ImageNet datasets: https://github.com/seshuad/IMagenet, accessed on 7 May 2025; CUMT-CMUID datasets and CUMT-HelmeT datasets: https://github.com/CUMT-AIPR-Lab, accessed on 14 May 2025.

## Abbreviations

The following abbreviations are used in this manuscript:

TENGs	triboelectric nanogenerators
NFC	near-field communication
ML	machine learning
DNN	deep neural network
DSC	depthwise separable convolution
MSA	mean squared activation
ADC	analog-to-digital converter
FLOPs	floating-point operations
